# Incremental value of enhanced plaque length for identifying intracranial atherosclerotic culprit plaques: a high-resolution magnetic resonance imaging study

**DOI:** 10.1186/s13244-023-01449-y

**Published:** 2023-05-25

**Authors:** XiaoQing Cheng, Jia Liu, HongXia Li, JiaLuo Yang, ChangSheng Zhou, BeiBei Zhi, QuanHui Liu, YingLe Li, LuLu Xiao, WuSheng Zhu, GuangMing Lu

**Affiliations:** 1 Department of Medical Imaging, Jinling Hospital, Affiliated Hospital of Medical School, Nanjing University, Nanjing, 210002 Jiangsu China; 2 Department of Medical Imaging, Jinling Hospital, The First School of Clinical Medicine, Southern Medical University, Nanjing, 210002 Jiangsu China; 3 Department of Medical Imaging, Jinling Hospital, Nanjing University of Chinese Medicine, Nanjing, 210002 Jiangsu China; 4 Department of Neurology, Jinling Hospital, The First School of Clinical Medicine, Southern Medical University, Nanjing, 210002 Jiangsu China; 5 Department of Neurology, Jinling Hospital, Affiliated Hospital of Medical School, Nanjing University, Nanjing, 210002 Jiangsu China

**Keywords:** MRI, Intracranial atherosclerotic, Plaque, Ischemic stroke

## Abstract

**Objectives:**

Besides plaque enhancement grade, the incremental value of enhancement-related high-resolution MRI features in defining culprit plaques needs further evaluation. This study was focused on assessing whether plaque enhancement features contribute to culprit plaque identification and further risk stratification.

**Methods:**

We retrospectively studied patients who experienced an acute ischaemic stroke and transient ischaemic attack due to intracranial atherosclerosis from 2016 to 2022. The enhancement features included enhancement grade, enhanced length, and enhancement quadrant. Associations between plaque enhancement features and culprit plaques, as well as diagnostic value, were investigated using logistic regression and receiver operating characteristic analyses.

**Results:**

Overall, 287 plaques were identified, of which 231 (80.5%) and 56 (19.5%) were classified as culprit and non-culprit plaques, respectively. Comparison of the pre- and post-enhancement images revealed enhanced length longer than the plaque length in 46.32% of the culprit plaques. Multivariate logistic regression showed that enhanced length longer than plaque length (OR 6.77; 95% CI 2.47–18.51) and grade II enhancement (OR 7.00; 95% CI 1.69–28.93) were independently associated with culprit plaques. The area under the curve value for the combination of stenosis and plaque enhancement grade for the diagnosis of culprit plaques was 0.787, which increased significantly to 0.825 on the addition of enhanced length longer than the plaque length (*p* = 0.026 for DeLong’s test).

**Conclusions:**

Enhanced length longer than the plaque length and grade II enhancement were independently associated with culprit plaques. The combination of the enhanced plaque features resulted in better culprit plaque identification.

**Supplementary Information:**

The online version contains supplementary material available at 10.1186/s13244-023-01449-y.

## Key points

- Enhanced length longer than the plaque length was independently associated with culprit plaques.
- Intraplaque haemorrhage and surface irregularities are common in plaques with enhanced length longer than the plaque length.
- The combination of enhanced plaque features enables better identification of culprit plaques.

**Supplementary Information:**

The online version contains supplementary material available at 10.1186/s13244-023-01449-y.

## Background

Intracranial atherosclerotic stenosis (ICAS) is one of the most important causes of ischaemic stroke worldwide, especially in Asian populations where ICAS accounts for more than half of all ischaemic strokes [1–3]. Plaque rupture with in situ thrombosis, which can cause an arterial–arterial embolism or arterial occlusion, is the main mechanism of stroke due to ICAS [4]. Moreover, histological studies have shown the presence of macrophage infiltration and increased neovascularisation in ruptured plaques, proving that inflammation plays a central role in atherosclerotic plaque breakdown [5]. High-resolution magnetic resonance imaging (HRMRI) studies of plaques have shown that plaque enhancement is associated with gadolinium leakage due to neovascularisation, active inflammation, and endothelial dysfunction, which can indirectly reflect the inflammatory state of the plaque and is independently associated with recent cerebrovascular ischaemic events, as well as stroke recurrence [6–8].

Currently, the grade of plaque enhancement is the most commonly used enhancement feature, with significantly enhanced plaques being associated with culprit plaques and stroke recurrence [9]. One study in which the most severe degree of plaque enhancement was used as an independent indicator, a moderate diagnostic ability for distinguishing the presence of acute cerebral infarction (area under the curve [AUC], 0.706; 95% confidence interval [CI] 0.625–0.787) was noted [10]. Furthermore, the persistence of plaque enhancement is a promising imaging marker for differentiating culprit plaques. Culprit plaques often exhibit the highest grade of baseline enhancement and persistent enhancement after follow-up [10]. A well-designed study could evaluate culprit plaques in follow-up. It has also been reported that culprit plaques can evolve under optimal medical treatment, in that plaque length and burden may reduce and there could be a decline in the degree of enhancement [11]. However, inconsistent results in the follow-up of culprit plaques may result from different follow-up intervals, different treatments, and data collected at different times of the patient's stroke onset. Therefore, the use of persistence of plaque enhancement to identify culprit plaques is controversial. Hence, this study was focused on assessing plaque enhancement features, including enhancement grade and quadrant and enhanced length, to determine the plaque enhancement features associated with culprit plaques in patients who experienced an ischaemic event and whether plaque enhancement contributes to the identification of culprit plaques and further risk stratification.

## Materials and methods

### Study patients

Patients with acute ischaemic stroke and transient ischaemic attack due to intracranial atherosclerosis who were admitted to our institution from November 2016 to June 2022 were included. The inclusion criteria were as follows: (1) age of > 18 years; (2) HRMRI performed within 8 weeks of symptom onset; (3) at least one intracranial atherosclerotic plaque identified on HRMRI and (4) ischaemic stroke confirmed by diffusion-weighted imaging (DWI) in the acute phase and by t2 fluid-attenuated inversion recovery imaging (FLAIR) and DWI in the subacute phase and chronic infarction. The exclusion criteria were as follows: (1) evidence of non-atherosclerotic intracranial vascular diseases, such as Moyamoya disease, artery dissection, or vasculitis; (2) extracranial carotid artery stenosis ≥ 50%; and (3) insufficient MRI quality. The clinical information recorded for each patient included sex, age, body mass index (BMI), hypertension, diabetes, hyperlipidemia, smoking, alcohol use, history of stroke and coronary heart disease, and laboratory data (triglycerides, total cholesterol, low-density lipoprotein, high-density lipoprotein, glycosylated haemoglobin, fasting plasma glucose, homocysteine).

### MRI examination

Imaging was performed using a 3.0 T whole-body MRI system (GE Discovery 750; GE Healthcare, Waukesha, Wisconsin, USA) with a 32-channel head coil. MRI protocols included 3D time-of-flight (TOF) magnetic resonance angiography, 2D high-resolution black-blood T2-weighted fast-spin-echo sequences, and pre- and post-contrast 3D high-resolution black-blood T1-weighted fast-spin-echo sequences, as well as conventional brain MRI. Enhanced T1WI was performed by obtaining repeated scans within 10 min after intravenous administration of gadopentetate dimeglumine injection (Beilu Pharmaceutical, Beijing, China) (0.1 mmol/kg, 2.0 mL/s). Detailed scan parameters are listed in Additional file 1: Table S1 Summery of imaging parameters.

### Image analysis

HRMRI scans were evaluated using an offline workstation (GE Healthcare 4.6). Image quality was assessed according to the following criteria: grade 0, where the external border and lumen of the artery could not be identified; grade I, where the external border and/or lumen were partially obscured; and grade II, where the lumen and external border were clearly defined, and the wall structures were visible in detail [12]. Only patients with grade II image quality were included in this study.

Culprit plaques were defined as (1) the only lesions within the vascular territory of the stroke or (2) the most stenotic lesions in the presence of multiple plaques within the same vascular territory [13]. A lesion was considered a non-culprit plaque if it was not within the vascular territory of the stroke. Any disagreements between the two reviewers in identifying the culprit plaque were resolved by discussion and consensus under the guidance of a senior neuroradiologist with 12 years of neuroradiological experience.

**Fig. 1:**
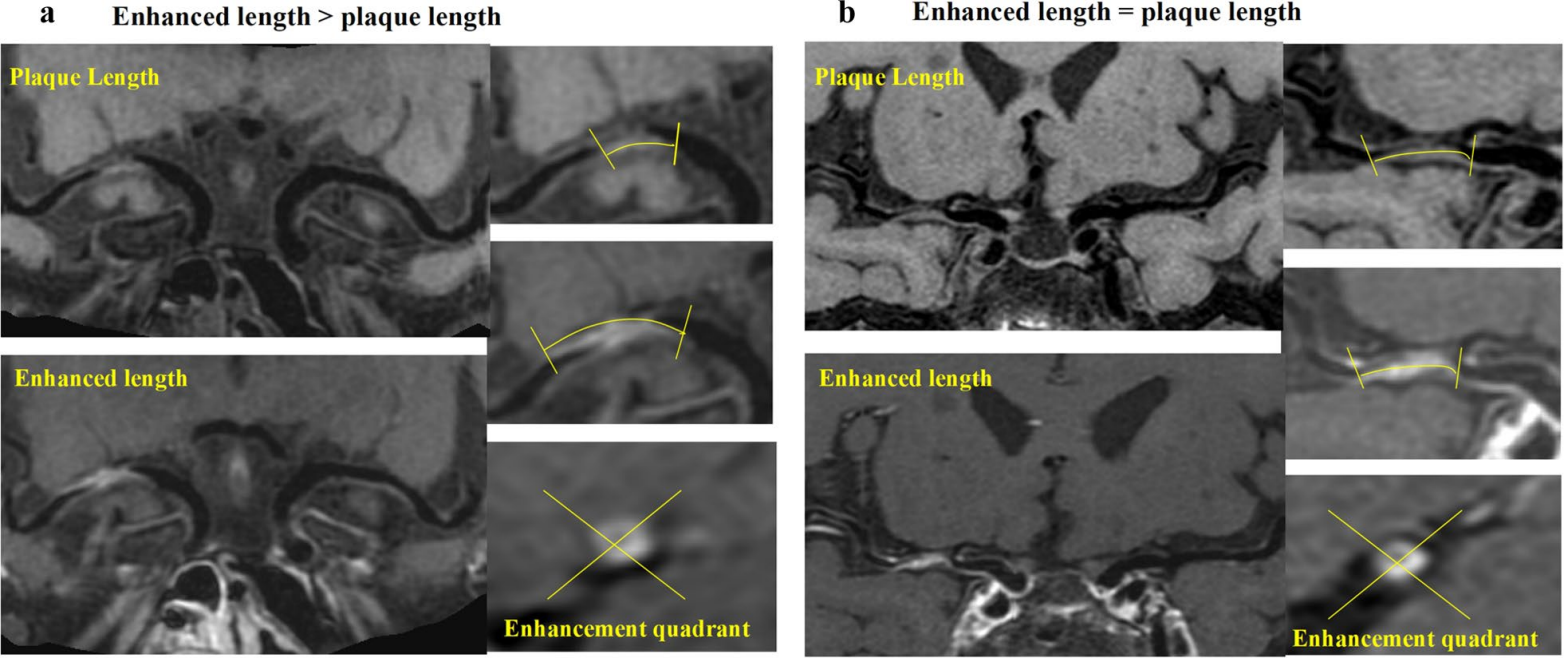
Measurements of plaque length, enhanced length, and enhancement quadrant. **a** Enhanced length > Plaque length; **b** enhanced length = plaque length

The clinical information of each patient was kept confidential when measuring plaque characteristics. Morphological features and signal characteristics of all plaques were determined independently by two neuroradiologists with 10 and 3 years of experience, respectively, and interobserver agreement was determined. The first neuroradiologist with 10 years of experience repeated the examination after 4 weeks to assess intra-observer agreement. The location of the plaque was divided into anterior and posterior circulation. The morphological characteristics of plaques measured included plaque length, plaque thickness, the degree of stenosis, plaque burden, remodelling index, and the surface of the plaque. Plaque length was measured from the proximal to the distal end of the plaque by using an image reconstructed with the curved planar reformation (CPR) technique (Syngo.via Research Frontier, MR Angio singleStation; Siemens Healthineers) (Fig. 1). Plaque thickness was measured at the site of the most stenotic lesion or the most apparent wall thickening on reconstructed images. The degree of stenosis was computed according to the Warfarin–Aspirin Symptomatic Intracranial Disease (WASID) study [14]. The degree of stenosis was divided into four grades (< 50%, 50–69%, 70–99%, and 100%). Both total wall area and lumen area were measured at the maximal stenosis site. The plaque burden at the site of most severe stenosis was calculated as follows: (total wall area − lumen area)/total wall area × 100% [15]. The remodelling index was defined as the ratio of the vessel area at the maximal lumen narrowing site to that at the reference site [16]. The reference site was selected based on the WASID study method. Remodelling indexes of ≥ 1.05, 0.95–1.05, and ≤ 0.95 were defined as positive, intermediate, and negative remodelling, respectively [16]. Plaque surface irregularity was defined as discontinuity of the plaque juxtaluminal surface [17].

Plaque signal characteristics include intraplaque haemorrhage, plaque enhancement grade, enhanced length, and enhancement quadrant. Intraplaque haemorrhage was defined as plaques with T1 hyperintensity and a signal intensity 1.5 times higher than that of the adjacent brain tissue or muscle [17]. Comparison of plaque enhancement features by using pre- and post-contrast 3D T1WI images. Plaque enhancement was graded using previously published grading criteria [13]: grade 0, no enhancement or enhancement similar to or less than that of the intracranial artery wall in the same individual without plaques; grade I, enhancement greater than grade 0 but less than that of the pituitary infundibulum; and grade II, enhancement similar to or greater than that of the pituitary infundibulum. Enhanced length was measured in the same way as plaque length, by using CPR-reconstructed post-enhancement images to measure the length of proximal to distal vessel wall enhancement (Fig. 1). The enhanced length was compared to the plaque length and divided into enhanced length > plaque length (Fig. 1a) and enhanced length ≤ plaque length (Fig. 1b). Cross-sectional images of plaques were divided into four quadrants [18], with counts based on the extent of the involvement of plaque enhancement (Fig. 1).

### Statistical analysis

Statistical analyses were performed using the statistical software package R (http://www.R-project.org, The R Foundation) and Empower-Stats (http://www.empowerstats.com, X and Y Solutions, Inc., Boston, MA). Categorical variables are presented as frequencies, and continuous variables are presented as means + standard deviations. The Kruskal–Wallis rank-sum test was used to test the differences between continuous variables, and the chi-square test was used to compare the differences between categorical variables. Logistic regression models were used to assess the correlation between plaque characteristics and culprit plaques. Model 2 was adjusted for sex, age, BMI, hypertension, and diabetes. Model 3, based on model 2, was further adjusted for intraplaque haemorrhage, plaque surface, enhancement grade, remodelling index, degree of stenosis, and plaque burden. Finally, receiver operating characteristic (ROC) curves were analysed for the identification of culprit plaques. Differences in the AUC values were assessed using the DeLong test. Reproducibility was evaluated using intraclass correlation coefficients (ICC). A *p* value of < 0.05 was considered statistically significant.

## Results

### Patient characteristics

**Fig. 2:**
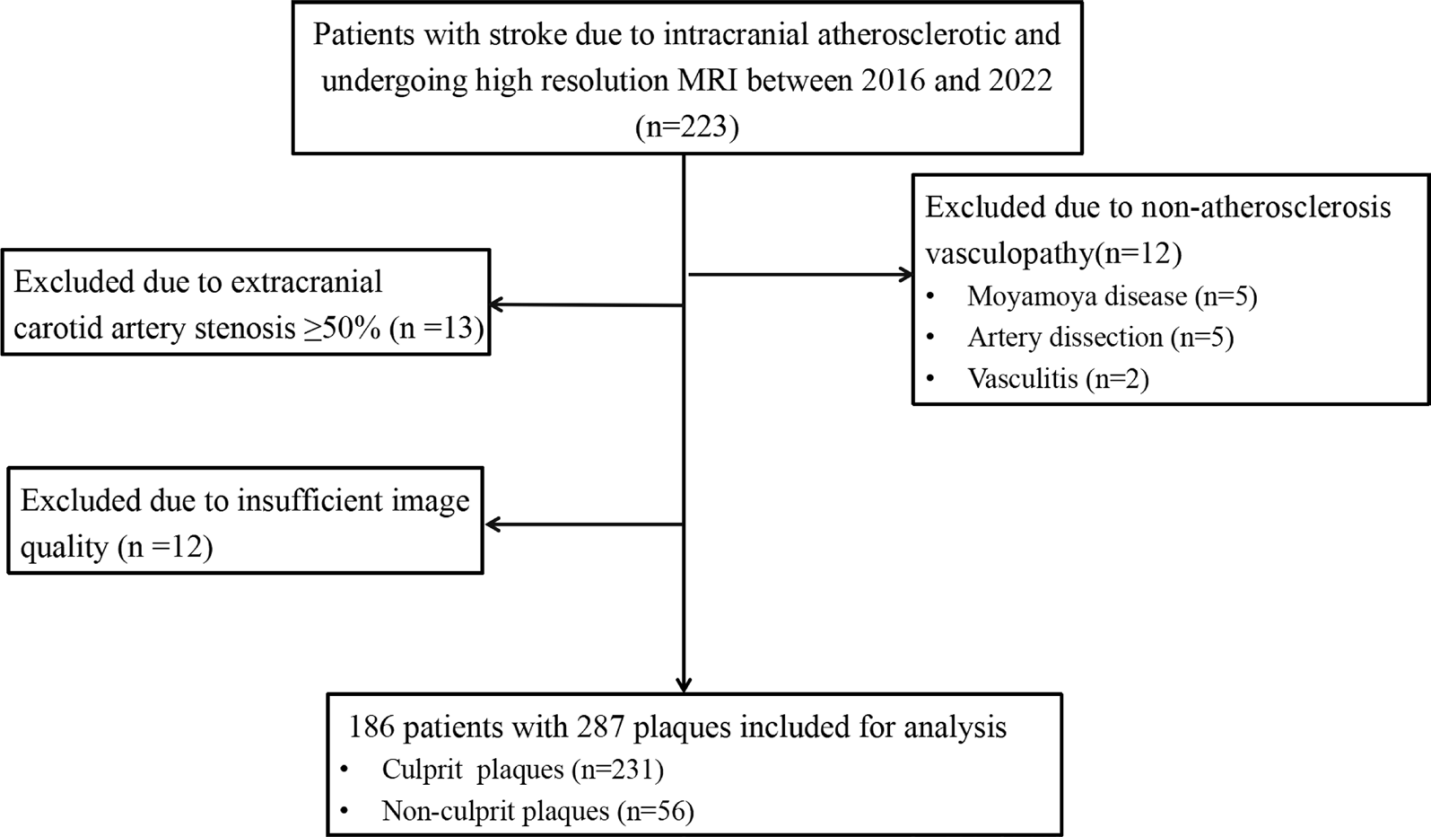
Flowchart of patient selection

Overall, 223 patients underwent HRMRI for acute ischaemic stroke or transient ischaemic attack during the inclusion period. Five patients with artery dissection, 5 with Moyamoya disease, 2 with vasculitis, and 13 with extracranial carotid artery stenosis ≥ 50% were excluded and so were 12 patients for whom images of sufficient quality were unavailable. In all, 186 patients (141 men and 45 women; mean age, 58.17 ± 11.50 years) were finally included for analyses (Fig. 2). Patient demographics and laboratory data are summarised in Additional file 1: Table S2 Demographic and clinical characteristics.

### Intracranial atherosclerotic plaque characteristics

In all, 287 intracranial atherosclerotic plaques were identified in the 186 patients. Of these, 190 (66.2%) and 97 (33.8%) were found in the anterior and posterior circulation, respectively. A total of 231 (80.5%) and 56 (19.5%) plaques were classified as culprit and non-culprit plaques, respectively. Compared with non-culprit plaques, culprit plaques had a higher prevalence of grade II enhancement (*p* < 0.001), intraplaque haemorrhage (*p* < 0.001), surface irregularity (*p* = 0.002), burden (*p* < 0.001), stenosis degree (*p* < 0.001), prevalence of enhanced length > plaque length (*p* < 0.001), and enhancement involving four quadrants (*p* < 0.001) and longer enhanced length (*p* = 0.001) (Table 1).

**Table 1:** Comparison of high-resolution MRI plaque features

	Non-culprit plaque (*N* = 56)	Culprit plaque (*N* = 231)	*p *value
Plaque length, (mm)	15.02 ± 8.08	16.66 ± 9.29	0.226
Plaque thickness, (mm)	1.89 ± 0.53	2.02 ± 0.68	0.208
Stenosis degree, (%)			< 0.001
< 50%	26 (46.43%)	46 (19.91%)	
50–69%	16 (28.57%)	42 (18.18%)	
70–99%	11 (19.64%)	97 (41.99%)	
100%	3 (5.36%)	46 (19.91%)	
Plaque burden, (%)	81.29 ± 10.34	88.44 ± 9.79	< 0.001
Remodelling index	1.01 ± 0.29	0.90 ± 0.30	0.009
Remodelling mode, (%)			0.158
Positive remodelling	20 (35.71%)	65 (28.14%)	
Negative remodelling	26 (46.43%)	139 (60.17%)	
Surface irregularity, (%)	12 (21.43%)	102 (44.16%)	0.002
Intraplaque haemorrhage, (%)	0 (0.00%)	45 (19.48%)	< 0.001
Enhancement grade, (%)			< 0.001
Grade 0, *n* (%)	6 (10.71%)	7 (3.03%)	
Grade I, *n* (%)	41 (73.21%)	73 (31.60%)	
Grade II, *n* (%)	9 (16.07%)	151 (65.37%)	
Enhanced length, (mm)	11.17 ± 7.70	15.95 ± 10.26	0.001
Enhanced length > plaque length, (%)	5 (8.93%)	107 (46.32%)	< 0.001
Plaque enhancement quadrant, (%)			< 0.001
0	5 (8.93%)	6 (2.60%)	
1	14 (25.00%)	15 (6.49%)	
2	11 (19.64%)	26 (11.26%)	
3	12 (21.43%)	38 (16.45%)	
4	14 (25.00%)	146 (63.20%)	

Mean + SD/*N* (%)

### Associations of plaque features with culprit plaques

Table 2 shows the results obtained using the univariate and multivariate logistic regression models. In the univariate analysis, the OR of the culprit plaque significantly increased with increasing enhanced length and grade. For the enhanced length, and grade, the ORs for tertile 3 were significantly greater than those for tertiles 1 and 2 (OR 3.11; 95% CI 1.40–6.90; *p* = 0.005 for enhanced length; OR 14.38; 95% CI 3.99–51.78; *p* < 0.001 for enhancement grade). In addition, stenosis degree greater than 70% (OR 4.98; 95% CI 2.27–10.95 for 70–99%; OR 8.67; 95% CI 2.45–30.65 for 100%), plaque burden (OR 1.07; 95% CI 1.04–1.10), plaque surface irregularity (OR 2.90; 95% CI 1.46–5.77), enhanced length (OR 1.06; 95% CI 1.02–1.10), enhanced length > plaque length (OR 8.80; 95% CI 3.39–22.85), and plaque enhancement involving four quadrants (OR 8.69; 95% CI 2.35–32.12) were positively associated with culprit plaques. Model 2 did not change the association between plaque characteristics and culprit plaque by adjusting for sex, age, BMI, hypertension, and diabetes. Model 3 was further adjusted for intraplaque haemorrhage, plaque surface, enhanced grade, remodelling index, and degree of stenosis, plaque burden on the basis of model 2, whereby multivariate regression analysis showed that enhanced length > plaque length (OR 6.77; 95% CI 2.47–18.51) and grade II enhancement (OR 7.00; 95% CI 1.69–28.93) remained associated with culprit plaques.

**Table 2:** Logistic regression analysis of plaque features associated with culprit plaques

	Non-adjusted OR (95% CI)	*p* value	Model 1 OR (95% CI)	*p* value	Model 2 OR (95% CI)	*p* value
*Plaque morphological features*						
Plaque length, (mm)	1.02 (0.99, 1.06)	0.226	1.03 (0.99, 1.07)	0.099	1.00 (0.96, 1.04)	0.926
Plaque thickness, (mm)	1.36 (0.84, 2.18)	0.209	1.56 (0.93, 2.62)	0.092	1.24 (0.66, 2.35)	0.501
Stenosis degree						
< 50%	1.0		1.0		1.0	
50–69%	1.48 (0.70, 3.14)	0.303	1.42 (0.65, 3.08)	0.381	0.62 (0.04, 9.25)	0.725
70–99%	4.98 (2.27, 10.95)	< 0.001	4.77 (2.15, 10.60)	0.001	1.44 (0.08, 27.32)	0.807
100%	8.67 (2.45, 30.65)	0.001	8.18 (2.28, 29.33)	0.001	0.84 (0.03, 24.96)	0.919
Plaque burden	1.07 (1.04, 1.10)	< 0.001	1.07 (1.04, 1.10)	< 0.001	1.02 (0.96, 1.08)	0.572
Remodelling index	0.29 (0.11, 0.74)	0.010	0.32 (0.12, 0.85)	0.023	0.29 (0.04, 1.91)	0.196
Remodelling mode						
Intermediate	1.0		1.0		1.0	
Positive remodelling	1.20 (0.50, 2.91)	0.680	1.32 (0.54, 3.24)	0.545	1.12 (0.41, 3.02)	0.830
Negative remodelling	1.98 (0.86, 4.58)	0.110	2.01 (0.85, 4.76)	0.111	1.24 (0.46, 3.37)	0.674
Surface irregularity	2.90 (1.46, 5.77)	0.003	3.24 (1.61, 6.54)	0.001	1.77 (0.80, 3.94)	0.159
*Plaque signal features*						
Enhanced grade						
Grade 0, *n* (%)	1.0		1.0		1.0	
Grade I, *n* (%)	1.53 (0.48, 4.85)	0.473	1.50 (0.47, 4.82)	0.492	1.31 (0.39, 4.46)	0.662
Grade II, *n* (%)	14.38 (3.99, 51.78)	< 0.001	13.34 (3.64, 48.92)	< 0.001	7.00 (1.69, 28.93)	0.007
Enhanced length, (mm)	1.06 (1.02, 1.10)	0.002	1.06 (1.03, 1.10)	0.001	1.03 (0.99, 1.07)	0.129
Enhanced length, (mm)						
Tertile 1, (5.13 ± 2.72)	1.0		1.0		1.0	
Tertile 2, (13.06 ± 2.57)	1.32 (0.67, 2.59)	0.417	1.37 (0.69, 2.72)	0.373	1.08 (0.51, 2.28)	0.835
Tertile 3, (26.65 ± 6.74)	3.11 (1.40, 6.90)	0.005	3.52 (1.56, 7.95)	0.003	1.81 (0.73, 4.50)	0.200
Enhanced length > plaque length	8.80 (3.39, 22.85)	< 0.001	9.10 (3.48, 23.83)	< 0.001	6.77 (2.47, 18.51)	< 0.001
Enhancement quadrant						
0	1.0		1.0		1.0	
1	0.89 (0.22, 3.59)	0.873	0.91 (0.22, 3.76)	0.899	0.74 (0.17, 3.24)	0.686
2	1.97 (0.50, 7.83)	0.336	1.76 (0.43, 7.22)	0.432	1.66 (0.38, 7.20)	0.501
3	2.64 (0.68, 10.21)	0.160	2.30 (0.58, 9.17)	0.238	1.70 (0.39, 7.49)	0.481
4	8.69 (2.35, 32.12)	0.001	8.00 (2.12, 30.16)	0.002	3.60 (0.83, 15.55)	0.086

Non-adjusted model. Model 1 was adjusted for sex, age, BMI, hypertension, and diabetes; Model 2 was adjusted for model 1 plus intraplaque haemorrhage, plaque surface, enhanced grade, stenosis degree, remodelling index, and plaque burden

### Association of plaque features with enhanced length > plaque length

Additional file 1: Table S3 (Logistic regression analysis of variables associated with Enhanced length > Plaque length) shows the univariate and multivariate logistic regression models used to verify the correlation between plaque characteristics and enhanced length > plaque length. In the univariate analysis, plaque burden, plaque surface irregularity, intraplaque haemorrhage, and degree of stenosis significantly correlated with enhanced length > plaque length. After adjusting for confounders, plaque surface irregularity (OR 1.88; 95% CI 1.06–3.35) and intraplaque haemorrhage (OR 2.24; 95% CI 1.03–4.88) remained independently associated with enhanced length > plaque length.

### ROC analysis

**Fig. 3:**
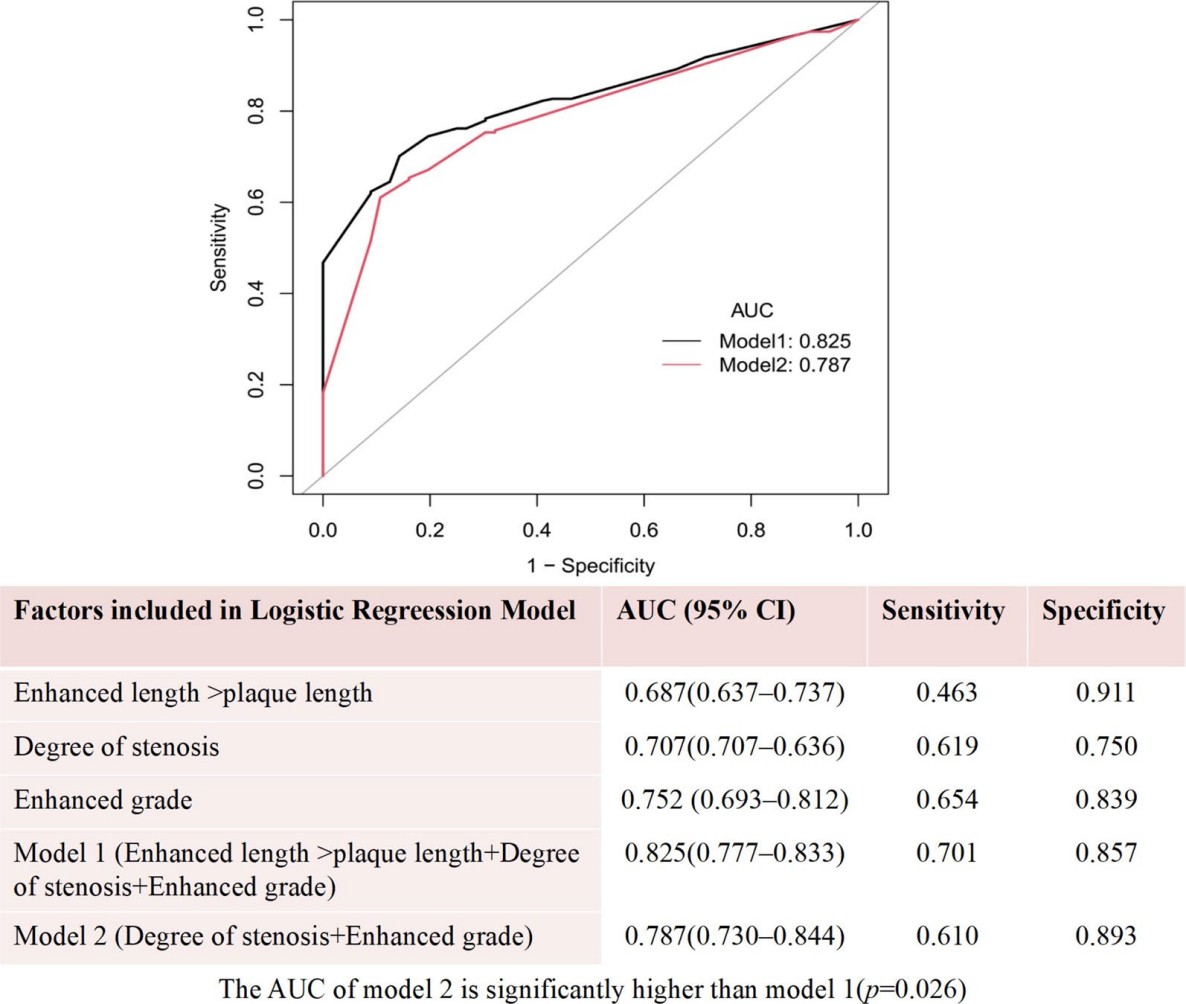
Receiver operating characteristic curves for identifying culprit plaques

The ROC analysis indicated that the AUC values for identifying culprit plaques were 0.707, 0.752, and 0.687, respectively, for degree of stenosis, enhancement grade, and enhanced length > plaque length. The AUC value for the combination of degree of stenosis and plaque enhancement grade was 0.787, and the addition of enhanced length > plaque length to the combination increased the AUC value to 0.825, with the sensitivity and specificity being 0.701 and 0.857, respectively. The DeLong test confirmed a statistical difference between the models (*p* = 0.026) (Fig. 3).

### Reliability of plaque feature measurements

There was good intrarater and interrater agreement (ICC, 0.887–1.000 and 0.802–1.000, respectively; Additional file 1: Table S4 The analysis of intra- and inter-observer reproducibility).

## Discussion

In this study, enhanced length > plaque length and grade II enhancement were significantly, independently, and positively associated with culprit plaques. Moreover, plaques with enhanced length > plaque length are more likely to have intraplaque haemorrhage and surface irregularities. The addition of enhanced length > plaque length to the combination of enhancement grade and degree of stenosis for the diagnosis of culprit plaque significantly improves the diagnostic efficacy.

Intracranial arteries have unique anatomical and physiological features. Physiological vasculature is present in the extracranially arteries, which can lead to enhancement on MRI under normal circumstances. In contrast, intracranial arteries lack vasa vasorum and the enhancement features of intracranial arteries may better reflect the pathological changes in plaques than the extracranial arteries [19]. In addition, intracranial arteries are surrounded by cerebrospinal fluid, lacking perivascular fat, and inflammation-related changes in perivascular fat attenuation reported in coronary and carotid artery-related studies could not be observed [20, 21]. Therefore, enhancement-related HRMRI features may be the most promising imaging markers reflecting the inflammatory response of intracranial plaques as well as plaque vulnerability.

We observed a significant positive correlation between enhanced length > plaque length and culprit plaques, with an adjusted ratio (OR 6.77; 95% CI 2.47–18.51), and similar results were obtained for grade II plaque enhancement (OR 7.00; 95% CI 1.69–28.93). Plaque enhancement is often considered a feature of the instability of atherosclerotic plaques and may be a predictor of plaque progression as well as stroke recurrence [8, 22]. Findings from the histopathological evaluation of carotid arteries suggest that plaque enhancement is associated with endothelial cell injury, vessel wall inflammation, and the result of neovascularisation [23]. In particular, studies have reported that a strong enhancement pattern was more prevalent in culprit lesions, which may indicate greater neovascularisation and/or inflammatory activity in these lesions, which in turn may indicate plaque vulnerability in culprit lesions [13, 24]. In addition, a study demonstrated that culprit plaque enhancement may persist for several months after an ischaemic event. The lack of enhancement at baseline or reduced enhancement at follow-up suggests that plaques are not the culprit [10]. The results of this study showed that 65.37% of the culprit plaques showed grade II enhancement and there was a significant positive correlation between the two. This result is similar to those of the previous studies mentioned above.

Interestingly, we found the signs of enhanced length > plaque length were significantly associated with a greater plaque burden, more frequent intraplaque haemorrhage, and irregularity of plaque surfaces, implying that this feature might serve as another marker of intracranial plaque instability and contribute to further risk stratification. According to previous studies, the AUC values for stenosis, plaque length, and plaque enhancement for the identification of culprit plaques ranged from 0.646 to 0.768 [10, 25, 26]. Kwee et al. [10] combined baseline and follow-up plaque enhancement to identify culprit plaques and the AUC value was 0.733. In the present study, in terms of differentiating the culprit plaque, grade II enhancement and enhanced length > plaque length were associated with comparable diagnostic efficacy (AUC, 0.752 vs. 0.687, *p* = 0.096) and both had a high specificity (0.911 vs. 0.839). The addition of enhanced length > plaque length significantly improved the efficacy of the combination of stenosis and enhancement grade in differentiating culprit plaques (AUC, 0.825 vs. 0.787, *p* = 0.026).

The enhanced length > plaque length feature was present in 46.32% of the culprit plaques in this study and the possible reasons are as follows: (1) When measuring plaque length on pre-contrast T1WI, there may be an underestimation of plaques with less thickness and poorly defined borders, whereas enhanced images provide excellent boundaries for measurements; and (2) some culprit plaques may have an inflammatory response in the vessel wall, resulting in enhancement of the vascular wall surrounding the plaque, resulting in the enhanced length being longer than the plaque length.

In this study, enhanced involvement of the four quadrants accounted for 63.2% of culprit plaques, which was significantly higher than that associated with non-culprit plaques. One study classified enhancement patterns as either type 1 (< 50% cross-sectional wall involvement) or type 2 (≥ 50% cross-sectional wall involvement). Type 2 enhancement is more prevalent in the case of culprit lesions and there is an independent correlation, suggesting that type 2 enhancement patterns may be a marker for more progressive and extensive plaques [24]. The type 2 enhancement pattern corresponds to the enhanced plaque involvement in quadrants three and four in our study. In the univariate analysis, plaque enhancement involving four quadrants was significantly associated with culprit plaques. However, the enhancement quadrant was not independently associated with culprit plaques when features were considered together.

The strength of this study is that it highlights that the feature enhanced length > plaque length when combined with plaque enhancement grade serves as a relatively simple and rapid method that is more suitable for clinical use than as a follow-up strategy to identify culprit plaques. However, several limitations should be noted. First, this was a single-centre retrospective analysis, which may have introduced selection bias into our results. Second, this study involved analysis at the plaque level rather than the patient level, and some of the culprit plaques were from the same patient as the non-culprit plaques and could not be correlated with circulating inflammatory biomarkers. Third, the lack of imaging-pathology comparisons in intracranial plaque imaging and pathological changes associated with plaque enhancement were inferred from hypotheses based on carotid and coronary artery-related studies.

## Conclusion

Enhanced length > plaque length and grade II enhancement were independently associated with culprit plaques in patients who recently experienced cerebrovascular ischaemic events due to intracranial atherosclerosis. Furthermore, the combination of both these features with the degree of stenosis had good discriminatory efficacy for determining culprit plaques. Further studies are required to evaluate the correlation between plaque enhancement features and inflammatory markers, which may be a contributing factor in future stroke recurrence.

## Supplementary Information

**Additional file 1. Table S1. **Summery of imaging parameters. **Table S2. **Demographic and clinical characteristics.** Table S3. **Logistic regression analysis of variables associated with Enhanced length > Plaque length. **Table S4.** The analysis of intra- and inter-observer reproducibility.

## Data Availability

The data that support the findings of this study are available upon request from the corresponding author. The data are not publicly available due to privacy and ethical restrictions.

## Abbreviations

AUC: Area under the curve
BMI: Body mass index
CPR: Curved planar reformatting
HRMRI: High-resolution magnetic resonance imaging
ICAS: Intracranial atherosclerotic stenosis
ICC: Intraclass correlation coefficients
