# Supplementary material for: Incremental value of enhanced plaque length for identifying intracranial atherosclerotic culprit plaques: a high-resolution magnetic resonance imaging study
Source: Insights Imaging. 2023 May 25;14:99. doi: 10.1186/s13244-023-01449-y (PMC10212900; doi:10.1186/s13244-023-01449-y)
Supplement: Supplementary file 1 — Additional file 1. Table S1. Summery of imaging parameters. Table S2. Demographic and clinical characteristics. Table S3. Logistic regression analysis of variables associated with Enhanced length > Plaque length. Table S4. The analysis of intra- and inter-observer reproducibility. [file 13244_2023_1449_MOESM1_ESM.pdf]

ELECTRONIC SUPPLEMENTARY MATERIAL

Incremental value of enhanced plaque length for identifying intracranial atherosclerotic culprit plaques: a high-resolution magnetic resonance imaging study

Supplemental Table 1: Summery of imaging parameters

Sequence	High-resolution MRI			Head MRI			
	3D T1WI	2D T2WI	3D TOF	Axial T1WI	Axial T2WI	DWI	FLAIR
Acquisition time	4min 57s	2min 37s	4min 13s	56s	57s	27s	56s
FOV (mm)	204×184	130×130	240×211	240×192	240×240	240×240	240×240
TR/TE (ms)	560/15.9	3400/57.6	20/3.4	1792/25.8	6361/105	3400/70	8400/118
Matrix	320 × 256	320 × 256	384×256	256×256	512×512	192×192	288×224
Slice thickness(mm)	0.8	2.0	1.4	5.0	5.0	5.0	5.0
Resolution	0.6×0.7×0.7	0.4×0.5	0.6×0.8×0.7	0.9×0.8	0.5×0.5	1.2×1.2	0.8×1.1
NEX	2	4	1	1	1	2	1
ETL	24	24	24	10	36	——	24
Flip angle (°)	90	111	15	111	142	90	160

DWI, diffusion-weighted imaging; ETL, echo train length; FLAIR, fluid-attenuated inversion recovery; FOV, field of view; TOF, time of flight; TR, repetition time; TE, echo time; NEX, number of excitations

Supplemental Table 2: Demographic and clinical characteristics (N =186)

	Results
**Patient Characteristics**	
Age, year	58.17 ± 11.50
Male, n (%)	141 (75.81%)
BMI, Kg/m2	25.02 ± 2.87
**Atherosclerotic risk factors**	
Hypertension, n (%)	148 (79.57%)
Diabetes, n (%)	71 (38.17%)
Dyslipidemia, n (%)	115 (61.83%)
Hyperuricemia, n (%)	29 (15.59%)
Homocysteine, n(%)	24 (12.90%)
Coronary heart disease, n (%)	14 (7.53%)
Smoke, n (%)	81 (43.55%)
Alcoholism, n(%)	55 (29.57%)
Stroke history, n(%)	44 (23.66%)
**Laboratory data**	
TG, (mmol/L)	13.47 ± 157.75
Total cholesterol, (mmol/L)	3.95 ± 1.72
LDL, (mmol/L)	2.18 ± 0.86
HDL, (mmol/L)	1.01 ± 0.25
FPG, (mmol/L)	6.01 ± 2.03
Glycosylated hemoglobin, (%)	6.54 ± 1.67
Homocysteine, (μmol/L)	13.45 ± 5.98

Note: Data are Mean+SD for continuous variables and N (%) for categorical variables. BMI, body mass index; TG, triglyceride; LDL, low-density lipoprotein; HDL, high-density lipoprotein; FPG,fasting plasma glucose.

Supplemental Table 3: Logistic regression analysis of variables associated with Enhanced length>Plaque length

	**Non-adjusted** OR (95%CI)	***P* value**	**Adjust I** OR (95%CI)	***P* value**	**Adjust II** OR (95%CI)	***P* value**
Plaque length	1.01 (0.98, 1.03)	0.575	1.01 (0.99, 1.04)	0.353	1.00 (0.97, 1.03)	0.838
Plaque thickness	1.37(0.95,1.97)	0.088	1.59 (1.07, 2.35)	0.020	1.35 (0.87, 2.08)	0.176
Stenosis degree						
<50%	1.0		1.0		1.0	
50%-69%	1.14 (0.52, 2.51)	0.739	1.18 (0.53, 2.61)	0.690	0.88 (0.38, 2.03)	0.756
70%-99%	2.79 (1.45, 5.35)	0.002	2.85 (1.47, 5.51)	0.002	1.56 (0.71, 3.42)	0.263
100%	3.39 (1.56, 7.36)	0.002	3.24 (1.48, 7.10)	0.003	1.66 (0.66, 4.18)	0.278
Plaque burden	1.05 (1.03, 1.08)	<0.001	1.05 (1.03, 1.08)	<0.001	1.03 (0.98, 1.09)	0.206
Remodeling index	0.59 (0.26, 1.33)	0.203	0.64 (0.28, 1.49)	0.305	1.33 (0.53, 3.33)	0.541
Remodeling mode						
Intermediate	1.0		1.0		1.0	
Positive remode	1.14 (0.50, 2.58)	0.760	1.11 (0.49, 2.54)	0.798	0.93 (0.35, 2.50)	0.892
Negative remode	1.54 (0.72, 3.26)	0.266	1.43 (0.66, 3.06)	0.363	1.17 (0.44, 3.09)	0.758
Surface irregularity	2.43 (1.49, 3.96)	0.004	2.55 (1.55, 4.20)	0.002	1.88 (1.06, 3.35)	0.031
Intraplaque hemorrhage	3.47 (1.78, 6.76)	0.002	3.36 (1.72, 6.55)	0.004	2.24 (1.03, 4.88)	0.042

Non-adjusted model adjust for: None; Adjust I model adjust for: sex,age,BMI,hypertension,diabetes; Adjust II model adjust for: sex, age, BMI,hypertension,diabetes, stenosis degree, plaque burden, remodeling index

Supplemental Table 4: The analysis of intra- and inter-observer reproducibility (Intraclass correlation coefficients)

**Plaque Features**	**Intra-observer**	**inter-observer**
Plaque length	0.894	0.823
Plaque thickness	0.985	0.900
Degree of stenosis	0.988	0.916
Plaque burden	0.983	0.802
Remodeling index	0.985	0.874
Surface irregularity	0.971	0.882
Intraplaque hemorrhage	0.940	0.867
Plaque enhancement grade	0.887	0.807
Enhanced length	0.995	0.845
Enhancement quadrant	1.000	1.000
